# Vaping-Induced Lung Injury With Superimposed Mycoplasma Pneumonia Leading to Acute Respiratory Failure

**DOI:** 10.7759/cureus.26755

**Published:** 2022-07-11

**Authors:** Humdoon Choudhry, Patrick Duplan

**Affiliations:** 1 Internal Medicine, Hospital Corporation of America (HCA) Florida Bayonet Point Hospital, Hudson, USA

**Keywords:** e-cigarette or vaping use-associated lung injury (evali), atypical pneumonia, respiratory failure, mycoplasma pneumonia, e-cigarettes, vaping pneumonitis, vaping

## Abstract

Electronic cigarettes (e-cigarettes) contain solvents, nicotine, and other chemicals which are vaporized with heat and inhaled into the lungs during a process known as vaping. Vaping has significantly increased in popularity in the US, especially among youth and young adults. E-cigarette, or vaping, product use-associated lung injury (EVALI) is a syndrome of lung disease associated with vaping or e-cigarette products--which is well discussed in the current medical literature. However, the mechanisms by which lung injury occurs remain to be fully understood. We hypothesize that vaping damages lung defenses, allowing bacterial or viral organisms to infect the lungs and further exacerbate lung function.

Furthermore, chemicals found in e-cigarettes alter lung structures, leading to an exaggerated response to an infectious insult. A combination of these two mechanisms may lead to acute respiratory failure. Here we discuss a case report about a 27-year-old patient who presented with acute respiratory failure due to vaping-induced lung injury with superimposed mycoplasma pneumonia.

## Introduction

Numerous cases of vaping-induced lung injury are reported in the medical literature as the use of vaping increases in the US [1-4]. One of the dangers of vaping is sudden and severe respiratory failure, known as E-cigarette, or vaping, product use-associated lung injury (EVALI). Typical symptoms of EVALI include severe shortness of breath, chest pain, cough, and fevers [2]. Currently, the criteria for diagnosis of EVALI include e-cigarette use in the last 90 days and exclusion of other etiologies that may be responsible for respiratory failure [5]. However, it is possible that due to vaping-induced damage to the lung’s natural defenses, microorganisms may infect and further contribute to oxygen impairment on top of already damaged and reduced lung function.

Studies have discussed that volatile chemicals in e-cigarette vapor might be responsible for damage to the lung’s epithelium layer and defenses, allowing bacterial or viral organisms to opportunistically better infect the damaged lungs [2-4,6]. In turn, a superimposed infection and vaping pneumonitis may cause patients to present with severe and sudden respiratory failure with much more exaggerated clinical symptoms than mycoplasma pneumonia or EVALI alone might. In addition, EVALI might not necessarily be a diagnosis of exclusion. Thus, clinicians should keep a high index of suspicion for concurrent infectious etiologies due to the risk of exponentially worsening respiratory failure. A multimodal approach to treating EVALI would lead to a better outcome. It would involve treating the lung injury and inflammation with steroids to allow the damaged epithelium to repair and with appropriate antibiotics to treat the superimposed infection if it is bacterial [7].

## Case presentation

**Figure 1:**
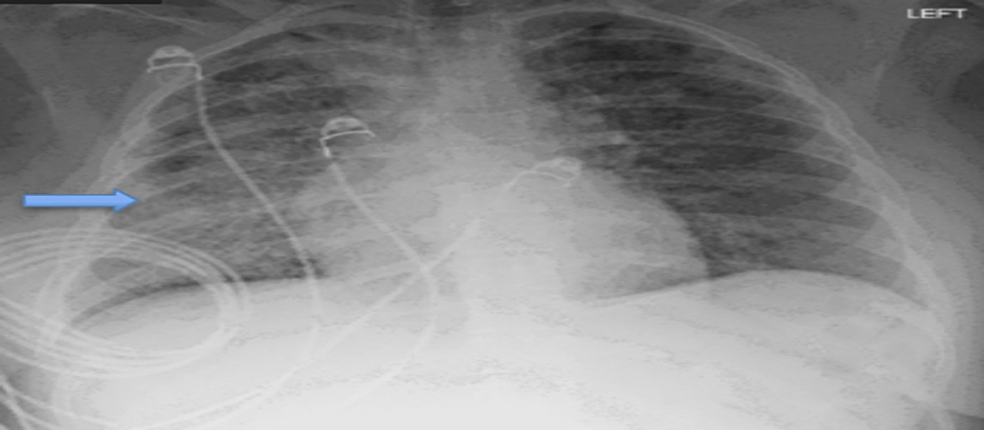
CXR upon admission. CXR shows lungs with bilateral interstitial infiltrates more extensive on the right (blue arrow) concerning for atypical pneumonia and pneumonitis.

A 27-year-old female with a history of anxiety disorder presented to our hospital with severe hypoxia. She was admitted to the intensive care unit (ICU) with acute hypoxic respiratory failure requiring noninvasive mechanical ventilation. The patient reported vaping daily as a coping mechanism for anxiety. Initial chest X-Ray (CXR) was concerning for bibasilar interstitial infiltrates (Figure 1).

**Figure 2:**
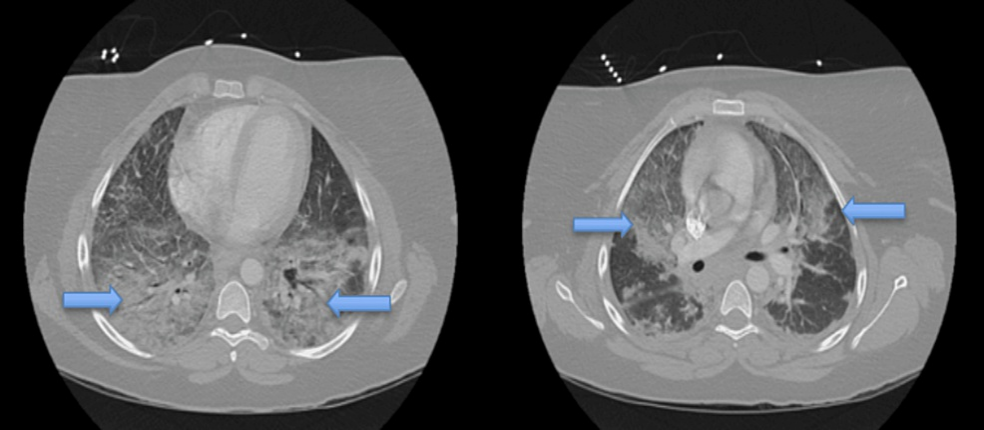
CT chest axial view CT chest axial views are remarkable for bilateral airspace opacities concerning EVALI. Blue arrows point to extensive opacities seen throughout the lung parenchyma. EVALI- E-cigarette, or vaping, product use-associated lung injury

**Figure 3:**
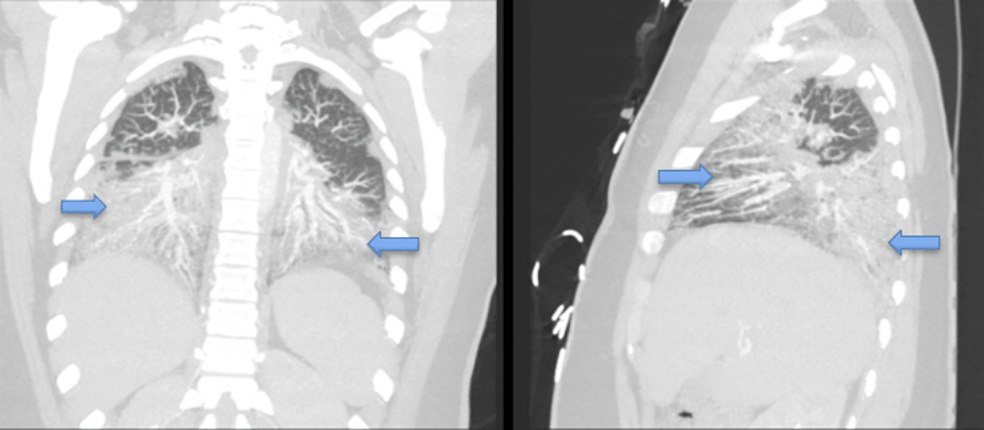
CT chest coronal and sagittal views CT chest coronal and sagittal views with bilateral airspace infiltration and opacities (blue arrows) related to infectious and inflammatory processes.

Computed tomography angiography (CTA) chest (Figures 2 and 3) was obtained and was remarkable for bilateral airspace infiltrates with suspected atypical pneumonitis. The patient’s COVID-19 rapid, Polymerase Chain Reaction test, Influenza A and B, and Legionella antigen were negative. However, she had elevated Mycoplasma pneumonia IgM titers. The patient’s workup included a pulmonology consult. The pulmonology service reviewed all the findings and had a high suspicion for EVALI even though she also had positive Mycoplasma pneumonia IgM Ab titers. Blood cultures were collected and were without any microorganismal growth.

The patient was treated with high-dose steroids (IV Methylprednisolone 125mg every 6hr for the first day, tapered down to 80mg every 8hr for the next two days, and then finally 40mg twice daily until the transition to oral Prednisone), inhaled Beta-adrenergic agonists and antibiotics (Oral Azithromycin 500 mg and IV Ceftriaxone 1gm daily). Daily arterial blood gas was obtained to assess her respiratory status and adjust the noninvasive mechanical ventilation settings. CXR was obtained every two days to assess lung damage and infection improvement. Initial repeat CXR showed worsening bibasilar interstitial infiltrates; however, the patient’s oxygen requirement for noninvasive ventilation and CXR started improving 48hr later. The patient was transitioned from noninvasive mechanical ventilation to supplemental oxygen towards the end of her ICU stay.

The patient’s length of stay in the ICU was six days, after which she was downgraded to the step-down unit. The patient had a lengthy stay in the ICU due to difficulty weaning her off the noninvasive mechanical ventilation after acute hypoxic respiratory failure. For the first three days, the patient was on noninvasive mechanical ventilation, after which she was gradually transitioned to a non-rebreather mask and eventually nasal cannula upon downgrade. The patient’s qSOFA (Quick Sequential Organ Failure Assessment) sepsis score during her ICU admission was 1. In addition, the patient had leukocytosis throughout her ICU stay. However, her leukocytosis was more due to steroid-related white blood cell demargination than infection since she was afebrile and without tachycardia. Finally, her ABGs were remarkable for metabolic alkalosis with respiratory compensation and low partial pressure of oxygen, which steadily improved after treatment with steroids and antibiotics.

**Figure 4:**
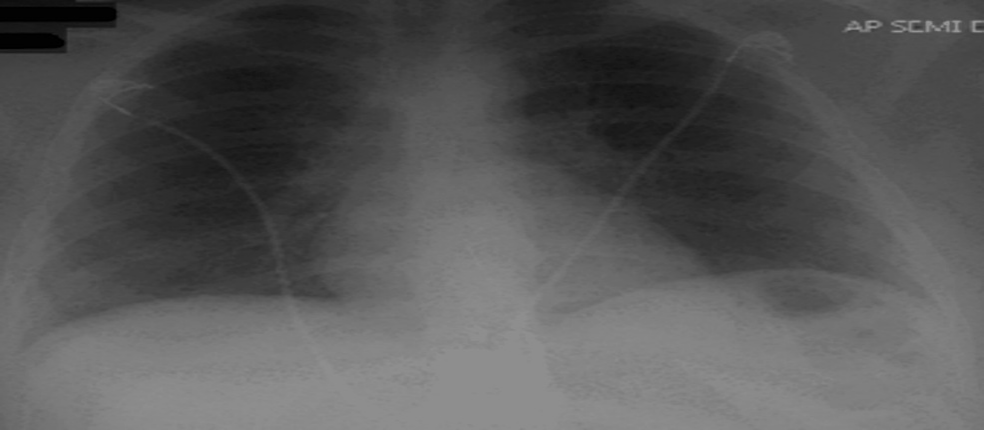
CXR with resolved bilateral opacities. Chest X-ray obtained the day before the patient was discharged shows a significant decrease in bilateral opacities and infiltrates after treatment with steroids and antibiotics.

A repeat chest X-ray upon downgrade from the ICU was significant for resolved bilateral opacities (Figure 4). The patient was discharged in stable condition on an extended course of oral Prednisone for a month and supplemental home oxygen via nasal cannula. The patient was also given outpatient follow-up with a pulmonologist to continue to monitor respiratory function.

## Discussion

Mycoplasma pneumonia is associated with atypical pneumonia, sometimes called ‘walking pneumonia’ since presenting symptoms are usually mild. Mycoplasma pneumoniae, the organism responsible for atypical pneumonia, is believed to impair the ciliary function of host cells and cause oxidative damage leading to inflammation [8]. Mycoplasma pneumoniae also becomes an opportunistic organism in those who are immunocompromised [8]. However, Mycoplasma pneumonia alone is typically not responsible for acute respiratory failure in young, healthy adults. Atypical pneumonia due to Mycoplasma pneumoniae tends to be benign unless the patient is elderly or immunocompromised [8]. Current literature has one reported case of EVALI with concurrent infection with Mycoplasma pneumonia leading to severe respiratory response [6]. Furthermore, Kooragayala et al. (2020) have discussed that vaping-induced injury increases the risk of viral and bacterial infections [6].

Our patient’s acute respiratory presentation hypothesis is two-fold: Cytotoxic vapor from e-cigarettes damages the lung’s defenses and epithelium, allowing either viral or bacterial organisms to infect the respiratory system and exacerbate lung function. Furthermore, any superimposed infectious pathology leads to an exaggerated inflammatory response due to vaping altered lung defensive and inflammatory response mechanisms. A combined effect of these mechanisms could lead to acute respiratory failure, as seen in our patient.

The mechanisms behind EVALI are poorly understood and need further investigation. The answer may lie with how the pulmonary cells respond to particular volatile chemicals inhaled during vaping, which may have severe cytotoxic pulmonary consequences. The Centers for Disease Control and Prevention lists certain chemicals in e-cigarettes, such as nicotine, propylene glycol, diethylene glycol, Acrolein, benzene, metals, and possible carcinogens [9]. Nicotine is believed to increase oxygen radicals, leading to point mutation in the DNA molecules and impairing mechanisms that develop and maintain lung structures [10]. Propylene glycol was found to alter circadian molecular genes in pulmonary cells, which may lead to an exaggerated immune response to bacterial organisms by altering endogenous cortisol release [11-12]. 

Similarly, Acrolein was found to alter inflammatory pulmonary response by increasing protease production, mucus secretions, and damaging epithelial cilia [13]. Benzene induced apoptosis and eventual necrosis in the epithelial cells, including rats’ bronchioles, terminal bronchioles, and alveoli [14]. Finally, metal exposure to pulmonary cells may lead to oxidative stress and increase inflammation-causing epithelial destruction and structural changes mimicking COPD [15].

These pathophysiological mechanisms point to two significant conclusions regarding vaping-related chemical exposure. Chemicals from vaping may damage the first-line defenses--the cilia and the integrity of the bronchioles and alveoli epithelial layer. In addition, there is an increase in the inflammatory response to the accumulation of cytotoxic substances, which possibly might not be cleared due to the damaged lung morphology and compromised mucociliary clearance. In light of these findings, viral or bacterial pathogens may easily infect the pulmonary cells. Due to an already heightened inflammatory response, an infectious insult may lead to respiratory failure.

Alexander et al. (2020) have explained that volatile vapors from e-cigarettes may cause damage to both alveolar epithelial cells and pulmonary endothelial cells leading to significant inflammatory response and eventual necrosis [16]. Due to damage to epithelial and endothelial cells, not only are the first-line defenses damaged, which may allow pathogenic organisms, such as Mycoplasma pneumonia, to infect the lung cells, but also inflammatory neutrophils and macrophages may accumulate and contribute to inflammation, possibly leading to an exaggerated response to pathogens [16].

## Conclusions

We discussed a case regarding an otherwise healthy young adult with a history of chronic vaping who presented with acute respiratory failure due to EVALI with superimposed Mycoplasma pneumonia infection. Only one case report has discussed EVALI with concurrent Mycoplasma pneumonia infection in the current medical literature. We discussed that EVALI alone might not necessarily be a different etiological reason for respiratory failure. It may present with an underlying infection or increased risk of developing a viral or bacterial infection due to damaged lung defenses. In addition, due to the accumulation of harsh chemicals found in the vapor of e-cigarettes, structural and inflammatory changes could contribute to respiratory failure seen in EVALI. EVALI diagnosis criteria might limit the treatment of patients who present with concurrent etiologies. Clinicians should have a high suspicion of concurrent etiologies and treat them accordingly.

## References

[1] Smith ML, Gotway MB, Crotty Alexander LE, Hariri LP (2021). Vaping-related lung injury. Virchows Arch.

[2] Crotty Alexander LE, Ware LB, Calfee CS (2020). E-cigarette or vaping product use-associated lung injury: developing a research agenda. an NIH workshop report. Am J Respir Crit Care Med.

[3] Park HR, O'Sullivan M, Vallarino J (2019). Transcriptomic response of primary human airway epithelial cells to flavoring chemicals in electronic cigarettes. Sci Rep.

[4] Christiani DC (2020). Vaping-induced acute lung injury. N Engl J Med.

[5] Abbara S, Kay FU (2019). Electronic cigarette or vaping-associated lung injury (EVALI): the tip of the iceberg. Radiol Cardiothorac Imaging.

[6] Kooragayalu S, El-Zarif S, Jariwala S (2020). Vaping associated pulmonary injury (VAPI) with superimposed Mycoplasma pneumoniae infection. Respir Med Case Rep.

[7] Siegel DA, Jatlaoui TC, Koumans EH (2019). Update: interim guidance for health care providers evaluating and caring for patients with suspected e-cigarette, or vaping, product use associated lung injury - United States, October 2019. MMWR Morb Mortal Wkly Rep.

[8] Lanao AE, Chakraborty RK, Pearson-Shaver AL (2022). Mycoplasma Infections. https://www.ncbi.nlm.nih.gov/books/NBK536927/.

[9] (2021). US Department of Health and Human Services. E-cigarette use among youth and young adults: a report of the Surgeon General. https://e-cigarettes.surgeongeneral.gov/documents/2016_sgr_full_report_non-508.pdf.

[10] Maritz GS (2008). Nicotine and lung development. Birth Defects Res C Embryo Today.

[11] Lechasseur A, Jubinville É, Routhier J (2017). Exposure to electronic cigarette vapors affects pulmonary and systemic expression of circadian molecular clock genes. Physiol Rep.

[12] Gibbs JE, Beesley S, Plumb J, Singh D, Farrow S, Ray DW, Loudon AS (2009). Circadian timing in the lung; a specific role for bronchiolar epithelial cells. Endocrinology.

[13] Moretto N, Volpi G, Pastore F, Facchinetti F (2012). Acrolein effects in pulmonary cells: relevance to chronic obstructive pulmonary disease. Ann N Y Acad Sci.

[14] Weaver CV, Liu SP, Lu JF, Lin BS (2007). The effects of benzene exposure on apoptosis in epithelial lung cells: localization by terminal deoxynucleotidyl transferase-mediated dUTP-biotin nick end labeling (TUNEL) and the immunocytochemical localization of apoptosis-related gene products. Cell Biol Toxicol.

[15] Rokadia HK, Agarwal S (2013). Serum heavy metals and obstructive lung disease: results from the National Health and Nutrition Examination Survey. Chest.

[16] Alexander LE, Bellinghausen AL, Eakin MN (2020). What are the mechanisms underlying vaping-induced lung injury?. J Clin Invest.

